# En-Bloc Resection of Stage T4 Non-Small Cell Lung Cancer with Direct Spinal Invasion: Technical Considerations and Comprehensive Literature Review

**DOI:** 10.3390/biomedicines14030733

**Published:** 2026-03-23

**Authors:** Wei-Ting Lee, Ke-Cheng Chen, Ching-Yao Yang, Yu-Cheng Yeh, Yen-Heng Lin, Yu-Cheng Huang, Jo-Yu Chen, Jin-Shing Chen, Fon-Yih Tsuang

**Affiliations:** 1 Division of Neurosurgery, Department of Surgery, National Taiwan University Hospital, No. 7, Chung-Shan South Road, Taipei 100, Taiwan; jimmyleesf@gmail.com; 2 Division of Thoracic Surgery, Department of Surgery, National Taiwan University Hospital, No. 7, Chung-Shan South Road, Taipei 100, Taiwan; 3 Department of Internal Medicine, National Taiwan University Hospital, No. 7, Chung-Shan South Road, Taipei 100, Taiwan; 4 Department of Orthopedic Surgery, Chang Gung Memorial Hospital, Linkou, No. 5, Fuxing St., Guishan Dist., Taoyuan 333, Taiwan; 5 Department of Medical Imaging, National Taiwan University Hospital, No. 7, Chung-Shan South Road, Taipei 100, Taiwan; 6 Spine Tumor Center, National Taiwan University Hospital, No. 7, Chung-Shan South Road, Taipei 100, Taiwan

**Keywords:** stage T4 lung cancer, en-bloc resection, spine invasion, R0 resection

## Abstract

Historically, stage T4 non-small cell lung cancer (NSCLC) with direct spinal invasion was considered a definitive surgical contraindication due to the perceived inability to achieve negative margins without catastrophic morbidity. This paradigm has shifted through the advancement of specialized surgical techniques, which facilitate radical en-bloc resection in highly selected candidates by adhering to the en-bloc concept. This concept mandates the retrieval of the tumor and invaded vertebral segments as a single, contiguous unit to prevent intralesional transgression and local recurrence. Achieving microscopic negative margins (R0) stands as the most critical prognostic factor, as radical resection offers a significantly improved potential for long-term survival. Technical success requires a meticulously planned multidisciplinary approach encompassing varied surgical corridors—ranging from combined anterior–posterior windows to single-stage posterior-only approaches—tailored to the tumor’s anatomical level. Furthermore, preoperative hemostatic optimization using dual-energy computed tomography (DECT) for vascular assessment and transarterial embolization (TAE) has become indispensable for managing the hypervascularity of the invaded vertebral bone. This review synthesizes these evolving strategies, illustrated by a case of a 74-year-old male with stage T4 NSCLC where an R0 resection was achieved through a two-stage approach integrating uniportal video-assisted thoracoscopic surgery (VATS). Ultimately, en-bloc management provides a feasible and potential surgical strategy toward long-term survival for localized, spine-invasive lung cancer within a multidisciplinary treatment framework.

## 1. Introduction

The concept of en-bloc resection originated in the management of musculoskeletal tumors, defined as the surgical removal of a lesion in a single, intact piece surrounded by a continuous layer of healthy tissue to avoid intralesional transgression [1]. This principle is further operationalized by the Weinstein–Boriani–Biagini (WBB) classification, which maps vertebral involvement across radial “layers”—extending from intraosseous components to the extraosseous paraspinal tissues [2]. While traditionally utilized for primary bone or soft tissue tumors originating within the vertebral body and invading outward, this en-bloc paradigm is increasingly applied to paraspinal malignancies with secondary osseous invasion. In these cases, the primary objective of en-bloc resection is the retrieval of the tumor and all involved osseous structures as an uninterrupted, contiguous unit to ensure microscopic negative margins (R0) [3].

For decades, locally advanced non-small cell lung cancer (NSCLC) with direct invasion of the vertebral body was considered a definitive contraindication to surgical resection. However, the pioneering work of Grunenwald and colleagues challenged this perspective, demonstrating that en-bloc resection involving total or partial vertebrectomy could be performed safely in highly selected candidates [4,5,6]. In the context of stage T4 spine-invasive tumors, achieving R0 resection often necessitates a total en-bloc spondylectomy (TES) to ensure the tumor remains untouched during the procedure [3,7]. This commitment to clear margins is the most critical prognostic factor, as incomplete resection (R1/R2) significantly compromises survival, while R0 status offers the potential for a long-term cure [8,9].

The technical complexity of en-bloc resection requires a meticulously planned, multidisciplinary approach. Surgeons must choose from various operative corridors tailored specifically to the tumor’s anatomical level and vascular involvement. Furthermore, because these tumors are often hypervascular, preoperative hemostatic measures such as transarterial embolization (TAE) have become indispensable to minimize intraoperative blood loss and maintain a clear surgical field [7,10]. These strategies collectively enhance the safety and feasibility of achieving a complete resection in a high-stakes operative environment.

Beyond the technical complexity, the management of Stage T4 NSCLC, which represents a highly heterogeneous group of patients, including those with superior sulcus (Pancoast) tumors and direct organ invasion, remains a subject of ongoing debate between clinical guidelines. This controversy is further compounded by the current lack of randomized controlled trials specifically addressing this population [11]. For clinical T4N0-1 disease, National Comprehensive Cancer Network (NCCN) guidelines suggest that either upfront surgical resection or neoadjuvant systemic therapy followed by surgery are viable options for resectable tumors [12]. Conversely, the Society of Thoracic Surgeons (STS) expert consensus emphasizes the role of induction therapy at high-volume centers, particularly recommending neoadjuvant chemoradiotherapy (CCRT) for superior sulcus tumors or those with spinal involvement to maximize the probability of an R0 resection [11]. Regarding more advanced T4N2 and T4N3 disease (Stage IIIB/C), the standard of care remains definitive CCRT followed by consolidation therapy [11,12]. While surgical intervention is generally discouraged in this setting, the STS consensus acknowledges its potential role within a multimodality framework for highly selected T4N2 subsets, such as those with satellite nodules in the same lobe [11]. Such discrepancies in international guidelines underscore the critical necessity of an individualized multidisciplinary team (MDT) approach to navigate these complex scenarios and optimize treatment outcomes.

We present a case of a 74-year-old male with stage T4 lung adenocarcinoma invading the T3 and T4 vertebral bodies who underwent successful multidisciplinary management. Following the case presentation, this review synthesizes existing surgical series and technical strategies to provide a comprehensive framework for managing these complex malignancies.

## 2. Case Presentation

### 2.1. Clinical History

**Figure 1:**
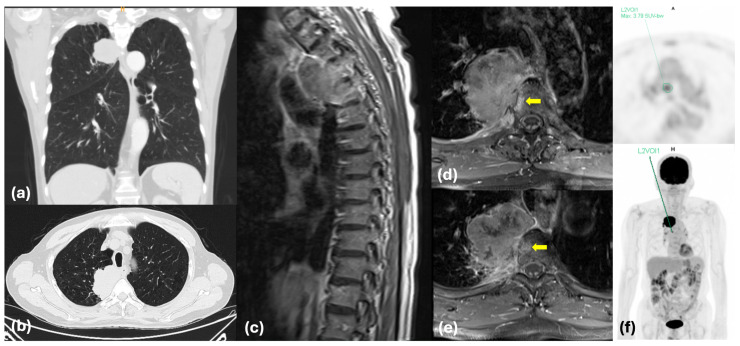
Preoperative radiographic and systemic staging of the patient. (**a**) Coronal view of the chest CT showing a 5.5 cm mass in the RUL. (**b**) Axial chest CT demonstrating direct invasion of the tumor into the mediastinum and vertebral column. (**c**) Sagittal T-spine MRI showing extensive involvement of the T3 and T4 vertebral bodies. (**d**) Axial MRI at the T3 level and (**e**) T4 level, both illustrating deep infiltration into the vertebral cancellous bone (arrow). (**f**) Whole-body PET scan and the localized precarinal lymph node; anterior (A); head (H).

A 74-year-old male presented with a four-month history of persistent right shoulder and upper back pain. The pain was intractable in the upright position and partially relieved when supine. Neurological examination revealed no focal motor deficits in all four extremities. His past history was unremarkable except for a remote gastric ulcer repair and appendectomy performed over 30 years prior. Initial radiographic evaluation via chest computed tomography (CT) revealed a 5.5 × 2.9 cm soft-tissue mass in the right upper lobe (RUL) with direct invasion into the mediastinum, pleura, and vertebral column (Figure 1a,b). Subsequent CT-guided biopsy confirmed pulmonary adenocarcinoma. Molecular analysis of the biopsy specimen revealed a wild-type EGFR status. Furthermore, PD-L1 expression was not evaluated as it was not part of the standard institutional diagnostic routine at the time of the patient’s initial presentation. Comprehensive systemic staging—comprising brain CT, whole-body positron emission tomography (PET), and thoracic spine magnetic resonance imaging (MRI)—showed no evidence of distant metastasis (Figure 1c–e); however, PET demonstrated equivocal uptake in the precarinal lymph nodes (Figure 1f), while thoracic MRI indicated extensive direct involvement of the T3 and T4 vertebral bodies. Given the patient’s intractable positional pain and a Spinal Instability Neoplastic Score (SINS) of 8, which categorized the spine as potentially unstable, immediate clinical intervention was prioritized. Consequently, further invasive mediastinal staging via endobronchial ultrasound (EBUS) was deferred to mitigate the imminent risk of neurological compromise from direct spinal invasion and to avoid critical treatment delay. The patient was clinically staged as cT4N2M0 (Stage IIIB). While the standard management for Stage IIIB disease typically involves neoadjuvant therapy followed by re-evaluation, the MDT prioritized immediate surgical intervention in this case. The patient’s clinical condition was severely compromised by intractable pain that rendered him bedridden, and his SINS of 8 indicated a potential risk of spinal collapse. Given that a conventional neoadjuvant course would necessitate an 8-to-10-week delay, the MDT concluded that such a waiting period posed a substantial risk of irreversible neurological deterioration. Furthermore, considering that the mediastinal involvement was limited to single-station, non-bulky lymphadenopathy, the team reached a consensus that upfront en-bloc resection and spinal stabilization, followed by a planned course of adjuvant therapy, represented the most appropriate strategy to achieve immediate symptom relief and optimize long-term oncological outcomes for our patient.

**Figure 2:**
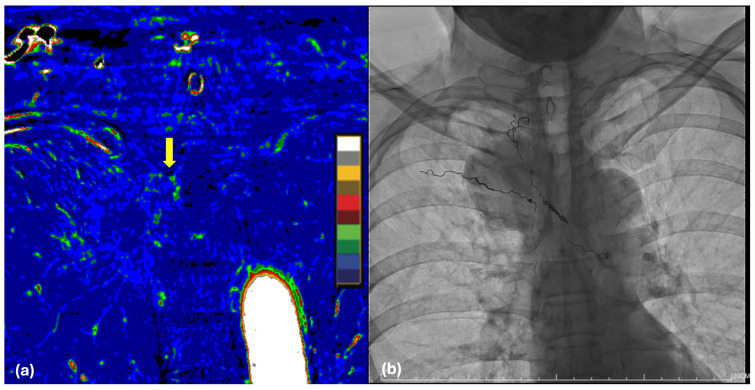
Preoperative hemostatic optimization and vascular assessment. (**a**) DECT demonstrating moderate hypervascularity within tumor (arrow). (**b**) Post-procedural radiograph following TAE showing the successful placement of embolization coils within the T3 and T4 intercostal.

Given the high vascularity inherent to the vertebral cancellous bone and intercostal arteries, coupled with the anticipated complexity of the total spondylectomy, a multidisciplinary preoperative protocol was implemented. Preoperative dual-energy CT (DECT) was utilized to evaluate regional vascularity, characterizing the tumor as a moderate hypervascular lesion (Figure 2a). To optimize intraoperative hemostasis, TAE of the bilateral intercostal arteries supplying the T3 and T4 levels was performed (Figure 2b). This targeted intervention was designed to minimize intraoperative hemorrhage and ensure a clear surgical field for the subsequent en-bloc resection. The success of the devascularization was later confirmed via specimen radiography, which demonstrated the embolization coils localized within the resected T3–T4 vertebral unit.

### 2.2. Two-Staged Surgical Procedure

The surgical strategy utilized a coordinated, two-stage approach involving both thoracic and spine surgical teams to ensure a true en-bloc resection.

#### 2.2.1. Stage 1: Posterior Spinal Release and Fixation

With the patient in the prone position, the spine surgeons performed posterior instrumentation from T1 to T6 using pedicle screws at the T1, T2, T5, and T6 levels. A wide laminectomy of T3 and T4 was performed, followed by T2/3 and T4/5 diskectomies. Bilateral T3 and T4 segmental arteries were identified and ligated. To facilitate en-bloc retrieval, the T3 and T4 vertebral bodies were circumferentially mobilized by meticulously releasing them from the aorta and esophagus, ensuring that oncological margins remained intact throughout the procedure.

#### 2.2.2. Stage 2: Thoracic Phase (VATS Lobectomy and Reconstruction)

**Figure 3:**
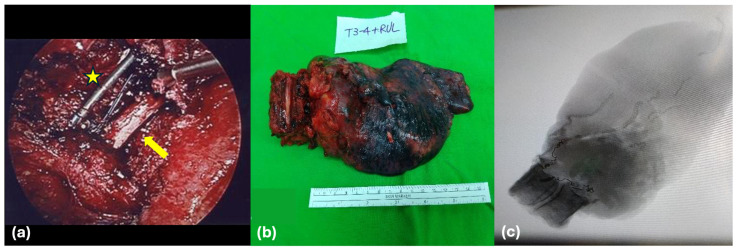
Intraoperative visualization and specimen analysis. (**a**) VATS view post-resection of the tumor showing the exposed spinal cord (arrow) and fixation rods (star). (**b**) Gross specimen of the lung tumor retrieved in continuity with the T3–T4 vertebral bodies. (**c**) Specimen radiograph confirming resection integrity and presence of embolization coils.

The patient was subsequently transitioned to the left lateral decubitus position for the thoracic approach. A uniportal video-assisted thoracoscopic surgery (VATS) was performed, during which the RUL was mobilized and a formal lobectomy was executed, alongside systematic mediastinal lymphadenectomy (Stations 3, 4, and 7). Crucially, the RUL was left attached to the T3 and T4 vertebral bodies to maintain the en-bloc integrity of the specimen. The entire complex was then retrieved through the thoracic incision as a single unit (Figure 3).

Following specimen retrieval, anterior spinal reconstruction was performed via the thoracic access. Under fluoroscopic guidance, an expandable titanium body cage was positioned within the T3–T4 defect to restore structural stability and sagittal alignment. After the placement of a chest tube for drainage, the patient was transferred to the intensive care unit for hemodynamic monitoring and postoperative management.

The total operative duration was 562 min. Estimated blood loss was 1000 mL during the posterior spinal release (Stage I) and 200 mL during the thoracic phase (Stage II).

### 2.3. Postoperative Course and Follow Up

The final pathology report confirmed a 4.8 cm adenocarcinoma with neuroendocrine differentiation. The tumor showed direct osseous invasion into the T3 and T4 bone. Lymph node analysis revealed one positive lobar node (1/4) and negative mediastinal nodes (Stations 3, 4, and 7 were 16/16 negative), resulting in a pathological stage of pT4N1M0. The surgical margins were microscopically clear (R0 resection). PD-L1 expression (Ventana SP263 assay) revealed a Tumor Cell (TC) score of 0%. Additionally, IHC testing for ROS-1 (D4D6 clone) and BRAF V600E (VE1 clone) both yielded negative results.

**Figure 4:**
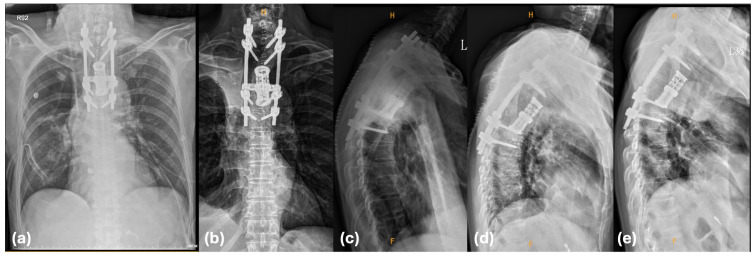
Postoperative radiographic surveillance. (**a**) Postoperative day 1 chest radiograph. (**b**) Anteroposterior (AP) radiograph at one month postoperatively demonstrating stable hardware positioning; Head (H); Foot (F). (**c**) Sagittal radiograph at one month, (**d**) two months, and (**e**) three months postoperatively confirming the maintenance of sagittal alignment and structural integrity of the expandable cage and posterior instrumentation.

The postoperative course was stable, and no new neurological deficit was noted. The hospital stay was prolonged to 24 days after surgery by postoperative pneumonia, which was successfully managed with targeted antibiotics. Subsequent surveillance, including CT and serial radiographs at one, two, and three months postoperatively, confirmed the structural integrity and stable positioning of the spinal instrumentation and expandable cage. There was no evidence of hardware migration, instrumentation failure, or loss of sagittal alignment throughout the follow-up period (Figure 4).

Due to the absence of actionable molecular targets, the patient was not a candidate for targeted therapy or immune checkpoint inhibitors. Consequently, he completed four cycles of adjuvant chemotherapy consisting of pemetrexed and cisplatin, followed by adjuvant radiation therapy. At the most recent clinical evaluation, six months postoperatively, there was no evidence of disease recurrence. However, the patient was subsequently lost to follow-up.

## 3. Literature Review

### 3.1. Evolution of Surgical Paradigms and Classification

Historically, the surgical management of NSCLC involving the spine was viewed as a definitive contraindication. For decades, vertebral body invasion was considered synonymous with irresectability due to the inability to achieve negative margins without risking catastrophic neurological or vascular damage [4,5,13]. This paradigm shifted following the landmark work of Grunenwald et al., who demonstrated that total or partial vertebrectomies could be performed safely en-bloc with the primary lung tumor [4].

Technical execution depends heavily on the epicenter of the tumor and the depth of invasion. Early experiences, such as those by Yokomise et al. and Aydinli et al., highlighted the efficacy of partial or hemivertebrectomies for lateralized tumors [14,15]. However, for tumors involving the vertebral body, TES has emerged as the definitive radical procedure [7].

To standardize surgical planning and patient selection, Fadel et al. proposed a classification system based on the depth of vertebral infiltration, which remains the foundational tool for determining the extent of osseous resection. In addition to resection strategies, Collaud et al. defined the principles for instrumentation and fixation based on the specific extent of resection required (Table 1) [3,6].

### 3.2. Technical Execution: The En-Bloc Concept

The fundamental principle of this surgery is the en-bloc concept, which mandates that the lung tumor and the involved vertebral segments be retrieved as a single, contiguous specimen. This approach stands in stark contrast to conventional techniques where the lung might be mobilized or peeled from the vertebral column before the bone resection. As established by Grunenwald et al. and Schirren et al., any maneuver that separates the tumor from the bone prior to removal is essentially intralesional [4,16]. Such piecemeal resections risk exposing the tumor core and exposing the surgical field to tumor seeding, which inevitably leads to local recurrence [10].

The technical difficulty of removing the lung and spine as one piece is justified by its superior local control. Maintaining the integrity of the tumor–vertebra interface ensures that the oncological “no-touch” principle is respected. Data from centers of excellence indicate that patients undergoing successful en-bloc resections with R0 margins achieve survival rates significantly higher than those where the lung and spine are removed separately [3,8,16].

### 3.3. Diagnostic Advancements and Preoperative Hemostatic Optimization

The MRI is the superior diagnostic tool for this planning phase, providing the most accurate assessment of invasion into the vertebral body and spinal canal—the ultimate factor in deciding operation strategy [3].

Furthermore, preoperative DECT has emerged as a significant non-invasive tool for assessing tumor vascularity, providing critical insights into intralesional blood flow [17,18,19]. Findings from DECT can guide the decision to perform preoperative TAE in cases with high vascularity. This intervention effectively mitigates the risk of massive hemorrhage from hypervascularized tumors, cancellous bone, and segmental or intercostal arteries, thereby ensuring a clearer surgical field for the execution of precise osteotomies [7,10].

### 3.4. Surgical Access: Combined vs. Single-Stage Posterior

Determining the optimal surgical access remains a point of significant academic discussion. The combined anterior–posterior approach has long served as the definitive benchmark for the management of complex stage T4 Pancoast tumors. As detailed by Fadel et al., this involves an anterior transcervical or thoracotomy window to release the thoracic inlet structures (subclavian vessels, brachial plexus) followed by a posterior approach for spinal stabilization [3]. This strategy ensures maximal control over anterior neurovascular structures, which is paramount in cases with extensive anterior infiltration (Table 2).

In contrast, the single-stage posterior-only approach has emerged as an increasingly utilized alternative, favored for its technical streamlining and procedural efficiency. As demonstrated by Wang et al. and Zairi et al., even complex stage T4 malignancies involving the posterior chest wall and vertebral bodies can be successfully resected en-bloc through a strictly posterior surgical corridor. This approach facilitates spinal stabilization, decompression, and the oncological resection within a single operative field. By bypassing the necessity for a secondary anterior phase, this method significantly reduces cumulative operative time and eliminates the physiological stress and potential positioning-related morbidity—such as airway displacement or increased anesthetic duration—associated with intraoperative patient repositioning required in combined approaches [10,13].

The current literature underscores that the selection of surgical access is strictly individualized. While posterior-only techniques continue to evolve, the combined anterior–posterior approach remains indispensable for cases requiring radical anterior clearance or the complex dissection of vital mediastinal structures—such as the great vessels and the pulmonary hilum—where such maneuvers are essential for ensuring oncological success. Regardless of the specific approach, the en-bloc concept is fundamentally defined by the achievement of an R0 margin.

### 3.5. Nodal Staging: The N2a/N2b Paradigm Shift

The most significant evolution in patient selection involves the interpretation of lymph node involvement. Historically, N2 disease was viewed as a definitive contraindication, as survival benefits were almost exclusively limited to N0/N1 patients [3,16].

However, the IASLC 9th Edition TNM classification has introduced a transformative sub-classification of N2 into N2a (single-station) and N2b (multi-station) (Table 3) [21]. Evidence cited by Kahya et al. demonstrates that N2a patients possess a significantly more favorable prognosis than those with N2b disease [22]. In specific T4N2 cases, such as those involving primary tumors under 3 cm with additional ipsilateral nodules, recent data suggest that surgery within a multimodal strategy may yield better survival outcomes than chemoradiotherapy alone [11]. Whether these findings imply that eligibility for en-bloc resection could be extended to include N2a patients remains a subject of debate, necessitating further prospective investigation across this heterogeneous disease spectrum.

### 3.6. Role of Induction Therapy

The use of induction therapy, whether delivered as neoadjuvant chemoimmunotherapy, radiotherapy or concurrent chemoradiotherapy (CCRT), is widely established as the cornerstone of multimodal management for stage T4 NSCLC with spinal involvement [23]. Its clinical significance is rooted in its role as a critical “biological filter” and prognostic indicator. Collaud et al. demonstrated that the pathologic response to induction therapy serves as an independent predictor of long-term survival [6]; a favorable response effectively suppresses micrometastases and significantly enhances 5-year survival rates. Furthermore, induction therapy is instrumental in improving R0 resection rates through effective tumor downsizing and the consolidation of the tumor–vertebra interface. This was quantitatively supported by Wang et al., who reported an 87.5% response rate following induction, which subsequently facilitated a high R0 resection rate of 83.3% [13]. These findings align with earlier observations by Yokomise et al. and Aydinli et al., who noted that induction protocols increase resectability while minimizing the risk of intraoperative tumor dissemination and strengthening local control through radiosensitization [14,15].

Drevet et al. reported a case series of 16 patients who underwent en-bloc resection for lung cancer invading the spine, with all receiving induction therapy prior to surgical intervention. In their cohort, the 3-year OS was 40% in the single-stage procedure group compared to 86% in the two-stage group. The authors concluded that while the resection of locally advanced NSCLC invading the spine is technically demanding and requires a high level of surgical expertise, favorable survival can be achieved following induction chemoradiotherapy, particularly when employing a staged surgical strategy [9]. Ultimately, induction chemoradiotherapy is highly effective in mitigating local recurrence risks, thereby ensuring the long-term oncological efficacy of radical en-bloc surgical intervention [10].

Despite the prevalence of induction protocols, a subset of the literature advocates for a “surgery-first” approach in selected cases to avoid treatment-related complications [3,7,16]. A primary concern is the escalation of surgical complexity; Fadel et al. noted that high-dose preoperative radiotherapy can induce significant tissue fibrosis, thereby obscuring anatomical planes and increasing the difficulty of radical dissection [3]. Similarly, Schirren et al. argued that while preoperative radiation (45–50 Gy) may offer limited local efficacy, it often compromises the integrity of the surrounding surgical field and impairs postoperative healing, leading them to favor a sequence of chemotherapy followed by surgery and subsequent radiotherapy [16]. Kuwata et al. also opted for direct surgical intervention to mitigate the risk of life-threatening spinal cord invasion or neoplastic meningitis, should the tumor fail to respond to induction therapy [7]. Furthermore, the perceived survival advantage of induction remains a subject of academic debate. Data from Fadel et al. indicated no significant difference in long-term survival between induction and non-induction cohorts [3]. This was further corroborated by a large-scale analysis of 135 cases, which demonstrated no statistically significant difference in survival outcomes between patients receiving induction therapy and those treated with primary surgery followed by adjuvant therapy [8].

With the evolution of precision medicine, the scope of induction therapy has expanded from traditional cytotoxic chemotherapy and radiation to include neoadjuvant targeted therapy and immunotherapy. For patients harboring EGFR mutations, the efficacy of neoadjuvant tyrosine kinase inhibitors (TKIs), specifically osimertinib, in promoting nodal downstaging and significantly increasing both major pathologic response (MPR) and R0 resection rates was recently demonstrated in the NeoADAURA trial [24]. Simultaneously, the advent of immune checkpoint inhibitors (ICIs) has transformed the therapeutic landscape of locally advanced NSCLC. Data from landmark trials such as CheckMate 816 have demonstrated that neoadjuvant chemo-immunotherapy significantly improves pathologic complete response (pCR) rates and event-free survival (EFS), establishing it as a standard option for resectable Stage IB to IIIA disease [23]. Furthermore, for unresectable T4 tumors, consolidation therapy with durvalumab following definitive chemoradiotherapy remains the established standard of care [12]. These contemporary oncological advancements underscore that treatment decisions for T4 lung cancer with spinal invasion must be informed not only by anatomical resectability but also by comprehensive molecular profiling and PD-L1 expression analysis, which are essential for guiding MDT consensus.

### 3.7. Oncological Outcomes

The current study demonstrates that long-term survival is attainable for stage T4 patients, provided a complete, en-bloc R0 resection is achieved. In major surgical series, the successful R0 rate ranges from 79–89% [6,8,13,20]. The 5-year overall survival (OS) rates for patients undergoing resection range from 31% to 52.5% [3,8,13]. More specifically, a systematic review by Collaud et al. highlighted that patients achieving an R0 resection demonstrated a superior 5-year OS rate of 48% (Table 4) [8].

Schirren et al. compared 20 surgical patients to 8 inoperable cases treated with definitive chemoradiotherapy. The non-surgical cohort showed a significantly shorter mean survival of 14.2 months compared to 46.0 months in the surgical group. With no survivors beyond 38 months, the 5-year overall survival for the inoperable group was effectively 0%, emphasizing that radical surgery remains the only reasonable path to long-term survival for stage T4 tumors invading the spine [16].

A pooled analysis confirmed that surgical margin status, specifically the distinction between R0 and R1/R2, stands as the only significant factor influencing long-term survival outcomes. The study revealed that patients with macroscopic residual disease (R2) or microscopic residual disease (R1) faced survival times that did not exceed 8 months and 41 months, respectively. These figures are substantially lower than the survival rates observed in R0 patients, underscoring that the presence of any residual tumor at the margin significantly compromises a patient’s prognosis compared to a complete resection where no malignant cells remain [8].

Local recurrence remains the primary challenge, particularly within the complex apical-vertebral anatomy where maintaining wide margins is technically demanding. To mitigate this risk, some centers integrate intraoperative brachytherapy or focused adjuvant radiotherapy along the bone resection margins to consolidate local control [25]. Ultimately, the high risk of specific complications—such as cerebrospinal fluid (CSF) leaks, respiratory failure, and hardware failure—underscores the necessity of performing these procedures in specialized centers with dedicated multidisciplinary expertise [16].

## 4. Discussion

### 4.1. The Oncological Necessity of the En-Bloc Concept in Vertebral Body Invasion

Historically, the management of stage T4 NSCLC with spinal invasion hinges on achieving complete negative margins. Historical data demonstrated that survival benefits are exclusively reserved for patients achieving an R0 resection; those with even microscopic residual disease (R1) experience survival outcomes comparable to non-surgical palliative care [3,16]. In our case, the preoperative MRI identified a vertebral body invasion, where the malignancy had breached the cortical bone to involve the cancellous marrow. Such deep infiltration renders hemivertebrectomy oncologically insufficient. The decision to perform a TES along with the lung tumor was predicated on the necessity of maintaining a wide, untouched margin around the tumor–vertebra interface. By retrieving the right upper lobe and the T3–T4 vertebral bodies as a single, contiguous unit, we avoided the risks of intralesional violation and tumor seeding inherent in piecemeal resections, ultimately securing the R0 status confirmed on pathology.

Furthermore, this en-bloc concept extends beyond thoracic malignancies; recent evidence from Pieters et al. regarding colorectal carcinoma with sacral invasion reinforces that achieving an R0 status through en-bloc sacrectomy significantly alters the prognostic trajectory [26]. Previous data demonstrated that failure to achieve a negative margin (R1 or R2 resection) resulted in a dismal median overall survival (OS) of only 7 months, whereas R0 resection extended survival to 23 months. Their multi-institutional data demonstrated that for properly selected Stage III (locally aggressive) patients, aggressive en-bloc management yielded a median overall survival exceeding 16 years. This underscores the core concept identical to our study: the absolute necessity of retrieving the malignancy and the invaded osseous structures as a single, contiguous unit to prevent intralesional violation and secure long-term survival.

### 4.2. Treatment Rationale

The management of patients with cT4N2M0 NSCLC and direct spinal invasion presents a profound clinical dilemma. Although current guidelines typically advocate for induction therapy as the standard of care for Stage IIIB disease, the clinical reality for patients with spinal involvement is often characterized by acute neurological symptoms or a high risk of imminent deterioration [12]. In these time-sensitive scenarios, adhering to a conventional 8-to-10-week neoadjuvant course may jeopardize the critical window for spinal cord decompression. Consequently, surgical decision-making must integrate not only oncological staging but also the SINS and the Epidural Spinal Cord Compression (ESCC) grade. When a high SINS indicates instability or an advanced ESCC grade suggests significant cord threat, upfront surgical stabilization and resection may be prioritized to prevent irreversible neurological deficit. This approach underscores the critical necessity of an MDT to effectively balance systemic oncological control against immediate local functional preservation.

### 4.3. Combined Approach and Utilization of VATS

Even though the usage of a single posterior approach has been recently popularized, we believe every case should be tailored. During our posterior approach, the negative margin can be achieved with direct visualization of the spinal cord, the nerve root, and the affected rib, the transverse process, and the costovertebral process. Often, the segmental artery at the contralateral side needs to be ligated. A posterior midline approach ensures this manipulation and fixation. During the anterior approach, a significant technical highlight of this case is the integration of uniportal VATS within a traditionally combined approach. Previously, VATS resection of a Pancoast was reported and discussed [27], but had not been discussed in combined surgery for en-bloc resection. While early pioneers like Grunenwald utilized extensive open thoracotomies or transcervical incisions for the anterior phase [4], the implementation of uniportal VATS facilitated minimally invasive, precise hilar mobilization and a formal systematic lymphadenectomy. This approach significantly reduced surgical trauma compared to a conventional large-incision thoracotomy. After dissection and ligation of the hilar structures, the en-bloc specimen can be removed via the VATS incision. Slight extension of the surgical incision was necessary to accommodate the removal of the large en-bloc specimen. We believe this strategy preserves the superior neurovascular control of the combined route, also avoiding the trauma of tissue during thoracotomy for complex stage T4 tumors, and leveraging minimally invasive techniques to accelerate postoperative recovery.

### 4.4. Hemostatic Optimization via Preoperative DECT and TAE

Historically, total spondylectomy in the thoracic spine is notoriously associated with high-volume intraoperative hemorrhage due to the rich vascularization of the tumor, the cancellous bone, and the segmental arteries. Achieving a dry surgical field is not merely a matter of safety but a prerequisite for the oncological precision required to maintain an untouched tumor–vertebra interface [7].

In our patient, the implementation of preoperative DECT allowed for a non-invasive, high-fidelity assessment of regional vascularity, characterizing the tumor as a moderately hypervascular lesion. This radiological insight directly informed the decision to perform TAE of the bilateral T3 and T4 intercostal arteries.

This interventional strategy provided two primary clinical benefits: it ensured a manageable 1200 mL blood loss during the aggressive spondylectomy phase and provided the clear field necessary to secure the R0 margins confirmed on final pathology. Furthermore, the presence of embolization coils within the resected T3–T4 unit, visualized on specimen radiographs, served as definitive proof of successful devascularization.

### 4.5. Surgical Efficiency and Hemostasis: Operative Duration and Blood Loss

Operative duration and intraoperative blood loss are critical metrics for evaluating the clinical feasibility and safety of radical en-bloc resections involving the vertebral column. In our case, the total operative duration was 562 min, which is comparable to the benchmarks established in the contemporary literature. The reported operative duration for the combined approach in the established literature ranges from 390 min [3,16] to 539 min [20]. Notably, while single-stage posterior approaches are often favored for their perceived procedural efficiency, evidence suggests that this approach does not significantly shorten operative time and may occasionally result in longer durations; for example, Wang et al. reported an average of 446 min [13], while Zairi et al. noted a mean duration of 540 min, with some cases extending up to 720 min [10]. This underscores that the inherent complexity of en-bloc resection remains the primary driver of operative duration, regardless of whether a single-stage or combined approach is utilized.

Regarding hemostatic control, our estimated blood loss of 1200 mL was substantially lower than the figures typically associated with these radical procedures. The literature for single-stage posterior resections indicates significantly higher blood loss, ranging from 2947 mL to 3200 mL [13]. Even in combined approach series, mean losses are reported around 2327 mL, with some instances exceeding 5400 mL [14]. We attribute our relatively limited hemorrhage to the proactive implementation of preoperative TAE and the use of uniportal VATS for precise, minimally invasive hilar mobilization. This synergistic multidisciplinary strategy ensured a clear surgical field, facilitating the oncological precision necessary to achieve the confirmed R0 status while minimizing perioperative morbidity.

### 4.6. Reconstruction Challenges

The mechanical instability resulting from a two-level total spondylectomy necessitates robust circumferential reconstruction. In our case, an expandable titanium cage was utilized to provide immediate anterior structural support, which is essential for maintaining sagittal alignment and preventing hardware failure. Historically, total vertebrectomy has required dual anterior and posterior stabilization, utilizing various materials such as autografts, titanium mesh or Pyramesh cages, and methylmethacrylate (Table 5) [4,13,15,16,20]. By integrating long-segment posterior fixation (T1–T6) to redistribute the biomechanical load away from the T3–T4 defect, this approach effectively precludes postoperative kyphosis and avoids instrumentation failure.

### 4.7. The Necessity of a Multidisciplinary Team Approach

The surgical management of tumors involving the vertebral body and thoracic cage necessitates a highly coordinated MDT. This collaboration bridges the expertise of thoracic surgeons, who manage pulmonary and vascular mobilization, with spine specialists responsible for vertebral resection and stabilization. Furthermore, the radiologist’s role has transitioned from simple diagnosis to active procedural support, which is essential for facilitating complex preoperative interventions. By utilizing DECT for precise preoperative mapping and performing TAE to devascularize the tumor, radiologists significantly optimize hemostasis. This integrated approach ensures that the pursuit of R0 margins is technically feasible while minimizing intraoperative blood loss and preserving the patient’s postoperative functional integrity.

## 5. Conclusions

En-bloc resection utilizing total spondylectomy for stage T4 NSCLC with direct spinal invasion represents a technically formidable but feasible paradigm. Adherence to the en-bloc principle in carefully selected patients—specifically the retrieval of the lung malignancy and the involved vertebral segments as a single, contiguous unit without prior separation—is essential to prevent intralesional seeding and ensure oncological integrity. Optimized through preoperative TAE and minimally invasive VATS techniques, this approach allows for radical R0 resections with manageable morbidity. Ultimately, an integrated multidisciplinary framework remains essential, not only for optimizing patient selection regarding treatment plan but also for the successful coordination of these complex, high-stakes interventions.

## Figures and Tables

**Table 1:** Anatomical classification of spinal invasion and required surgical reconstruction.

Classification	Anatomical Depth of Invasion	Surgical Strategy	Instrumentation
Type I (TP group)	Transverse process (TP)/ Costovertebral angle	Transversectomy/ Partial Vertebrectomy	Not typically required
Type II (IF group)	Intervertebral foramen (IF)	Hemivertebrectomy	Posterior stabilization often required
Type III (VB group)	Cancellous bone of the vertebral body (VB)	Total En-Bloc Spondylectomy (TES)	Posterior Stabilization + Anterior Reconstruction

**Table 2:** Summary of surgical access strategies and clinical highlights based on tumor location; posterolateral (PL); lateral decubitus (Lat. debuc.).

Tumor Location	Approach	Position	Source Study	Highlight
Pancoast (C8-T3)	Posterior	Prone	Wang et al. [13] Zairi et al. [10]	Avoid repositioning
	Anterior + Posterior	Supine ⟶ Prone	Fadel et al. [3] Novellis et al. [20]	Better thoracic inlet neurovascular control
Non-Pancoast (Below T4)	Posterior + PL Thoracotomy	Prone ⟶ Lat. decub.	Aydinli et al. [15] Schirren et al. [16]	Optimal circumferential exposure
	PL Thoracotomy	Supine ⟶ Table rotation	Yokomise et al. [14]	Not suitable if subclavian vessel involved
Thoracolumbar (T11-L2)	PL Thoracotomy + Posterior	Lat. decub. ⟶ Prone	Novellis et al. [20]	Via left side to avoid liver injury

**Table 3:** Evolution of nodal staging and surgical eligibility (IASLC 8th vs. 9th Edition).

N-Descriptor	8th Edition Category	9th Edition Category (2025) [12]	Prognostic Implication	Surgical Eligibility
N0/N1	N0 or N1	N0 or N1	Favorable	Primary candidates
Single-station N2	N2	N2a	Significantly better than N2b	If non-bulky tumor and adequate patient condition [22]
Multi-station N2	N2	N2b	Poor	Relative contraindication

**Table 4:** Summary of key surgical series for stage T4 NSCLC with spinal invasion.

Source Study (Year)	Study Design	Total Patients (*n*)	Induction Therapy Rate (%)	Operative Approach	Complete Resection (R0) Rate	5-Year OS (Total Cohort)	5-Year OS (R0 Subgroup)
Grunenwald (1996) [4]	Cohort	19	58.0%	Combined	NA	14.0%	NA
Collaud (2013) [6]	Cohort	48	100.0%	Combined/ Posterior	88.0%	61.0%	69.0%
Collaud (2015) [8]	Systemic review	135	63.0%	Combined/ Posterior	89.0%	43.0%	48.0%
Wang (2022) [13]	Cohort	18	89.0%	Posterior	83.3%	52.5%	NA
Novellis (2023) [20]	Cohort	16	62.5%	Combined/ Posterior	79.0% *	20.0%	NA

* Including primary vertebral tumor and metastasis patient.

**Table 5:** Comparison of materials used for anterior spinal reconstruction.

Source Study (Year)	Material	Approach	Characteristics
Grunenwald (1996) [4]	Autogenous clavicle graft	Combined (Anterior + Posterior)	Convenient local autograft harvested during anterior approach, eliminates second donor site and promotes biological fusion
Schirren (2011) [16]	Titanium mesh cage + bone graft	Combined (Posterior + PL Thoracotomy)	Mesh cage in conjunction with bone grafting, ventral osteosynthesis and anterior vertebral body fixation plate
Aydinli (2004) [15]	Methylmethacrylate (bone cement) filled in chest tube	Combined (Posterior + PL Thoracotomy)	“Chest-tube technique” used to mold cement into a patient-specific strut
Wang (2022) [13]	Pyramesh cage (titanium cage)	Single-Stage Posterior	Expandable design allows posterior insertion and anterior column support within a single-stage procedure
Novellis (2023) [20]	Vertebral body replacement cage filled with autograft	Mixed (Combined/Single)	Provides immediate structural support while internal autograft promotes durable biological fusion

## Data Availability

No new data were created or analyzed in this study.

## Abbreviations

The following abbreviations are used in this manuscript:

DECT	dual-energy computed tomography
TAE	transarterial embolization
VATS	video-assisted thoracoscopic surgery
LD	non-small cell lung cancer
TES	total en-bloc spondylectomy
IASLC	International Association for the Study of Lung Cancer
CT	computed tomography
PET	positron emission tomography
MRI	magnetic resonance imaging
MDT	multidisciplinary team
RUL	right upper lobe
TP	transverse process
VB	vertebral body
IF	intervertebral foramen
PL	posterolateral
Lat. decub	lateral decubitus
CCRT	concurrent chemoradiotherapy
Gy	gray
OS	overall survival
